# Safe prescribing training provision for junior doctors: is this optimal?

**DOI:** 10.1186/s12909-016-0748-4

**Published:** 2016-08-24

**Authors:** Maria B. Kennedy, Muzaffar Malik, Inam Haq, Sian E. Williams, Michael Okorie

**Affiliations:** 1 Division of Medical Education, Brighton and Sussex Medical School, Brighton, United Kingdom; 2 Medical Statistics, Division of Medical Education, Brighton and Sussex Medical School, Brighton, United Kingdom; 3 Sydney Medical Program, Office of Medical Education, University of Sydney, Sydney, Australia; 4 Health Psychology, School of Pharmacy and Biomolecular Sciences, University of Brighton, Brighton, United Kingdom; 5 Medicine and Medical Education, Division of Medical Education, Brighton and Sussex Medical School, Brighton, United Kingdom

**Keywords:** Safe prescribing, Practical prescribing, Prescribing errors, Postgraduate medical education, Foundation programme training

## Abstract

**Background:**

The aim of this study was to determine the training provisions in practical safe prescribing for foundation doctors in NHS hospitals located in the South Thames region.

**Methods:**

A web-based questionnaire was distributed by e-mail to all 1762 foundation doctors in the South Thames Foundation School (STFS) region. In addition, a separate questionnaire was distributed to prescribing training Leads at 15 NHS Hospital Trusts. Quantitative data were analysed using descriptive statistics and thematic analysis was performed on qualitative data.

**Results:**

Trainers: 10 Prescribing Leads (67 %) responded. Of the 9 NHS Trusts that offered safe prescribing training in their induction programme, 5 included a practical prescribing session. By the end of the foundation year, 6 NHS Trusts had provided at least one dedicated practical prescribing session for F1s compared with 2 NHS Trusts for F2s.

Trainees: A total of 124 foundation trainees (7.2 %) responded (69 F1s and 55 F2s). 87 % of F1s received dedicated training in safe prescribing at their Trust induction (*n* = 60) in comparison to 49 % of F2s (*n* = 27). 80 % of F1s (*n* = 55) had a practical prescribing session during induction versus 27 % of F2s (*n* = 15). The difference was significant, *X*^*2*^ (1, *N* = 124) = 34.23, *p <0.0001.* Emerging themes from qualitative data included, recognition of medical education as a continuum, importance of working relationships with pharmacists and neglect of F2s.

**Conclusions:**

There appears to be a lack of emphasis on the training of F2 doctors in practical safe prescribing compared with F1 doctors. There should be standardisation of safe prescribing training provisions, particularly in the induction period and for F2 doctors.

**Electronic supplementary material:**

The online version of this article (doi:10.1186/s12909-016-0748-4) contains supplementary material, which is available to authorized users.

## Background

Prescribing medicines is one of the most common and important forms of therapeutic intervention made by doctors in both the primary and secondary care setting [1]. Despite its obvious importance and the frequency at which prescribing is performed, it remains an error-prone process, which can compromise patient safety. It has been recognised that the process of prescribing does not just entail putting pen to paper, but originates from the therapeutic decision to use a drug in clinical practice. Prescribing errors can occur during either of these processes [2] and efforts to maintain a high standard of prescribing are essential.

The EQUIP study, highlighted the prevalence and nature of prescribing errors by foundation doctors in the UK [3]. Researchers found a mean error rate of 8.9 % across all grades of doctors. Foundation doctors were responsible for the highest error rates, and unsurprisingly performed the majority of prescribing. Doctors in their second year of postgraduate training (F2s) were found to have the highest error rate at 10.3 %, followed by doctors in their first year of postgraduate training (F1s) at 8.4 % and the reasons for this are yet to be explored fully. Prescribing error is not a problem exclusive to the hospital setting, as 4.9 % of prescriptions written in general practice, in the UK, contain an error [4].

Notably, new graduates do not feel adequately prepared to undertake the complex task of prescribing [5]. One possibility is that attempts to optimise the undergraduate medical curriculum in line with General Medical Council (GMC) guidance [6] have inadvertently created a void in terms of knowledge acquisition and prescribing skills in pharmacology and therapeutics. The UK Foundation Programme Curriculum 2012 [7] specifies a list of competencies that must be achieved but autonomy is afforded each NHS trust regarding how they ensure their trainees meet the learning outcomes and demonstrate themselves to be proficient practitioners.

Although there is a body of evidence to suggest that junior doctors’ confidence increases with greater exposure, familiarity and knowledge [8, 9], this does not eliminate error completely and conversely may result in an ill-placed sense of confidence amongst more experienced F2s. It is unknown to what extent, if any, this may contribute to the higher prescription errors rates observed amongst this group of doctors.

Dornan et al. concluded that the paucity of training in practical prescribing coupled with doctors’ unfamiliarity with drug charts are both contributing factors that can bring about prescribing errors. It was recommended that training in practical prescribing be offered to all F1 doctors, and furthermore, highlighted its importance during induction [3]. This might address the gap between theory and practice. One shortcoming of this however is that the recommendation was made for F1 training only, despite F2s having the highest prescribing error rate.

To date, reports investigating the training provisions in practical safe prescribing for junior doctors are lacking. This study sought to identify the level of provision of practical prescribing training for F1 and F2 doctors in NHS hospitals located in the South Thames Foundation School (STFS) region. The STFS oversees the training of over 1700 F1 and F2 doctors in a large geographical area in the South East of England in the UK [10].

## Methods

### Design

An e-questionnaire was utilised to elicit information regarding safe and practical prescribing training provisions during the period of August 2013 and July 2014, with the induction period and the remainder of the foundation-training year scrutinised. This was in an attempt to ascertain the training provided and which aspects, if any, were mandatory. In addition, information was obtained on which sessions the respondents perceived to be useful. The questionnaire was available for completion between April and July 2014. A mix of open and closed questions was used in the study.

### Respondents and setting

F1 and F2 doctors (trainee), as well as individuals responsible for providing prescribing training (trainers) at the NHS Hospital Trusts in the region were invited to participate in the study via e-mail. Participants were identified via the local Health Education England organisation, the STFS and the postgraduate centres at the various hospitals. A link to the trainee questionnaire was sent out in online bulletins both from the STFS and at some Trusts that utilise e-communications. In addition, each NHS Trust’s postgraduate medical centre was contacted directly by the research team and asked to forward a link to the survey via e-mail to their junior doctors. Prescribing leads were contacted directly via e-mail. The foundation school and the postgraduate medical centres sent out reminder e-mails on behalf of the research team.

### Questionnaire design and administration

Questionnaires (Additional files 1 & 2) were designed using the Bristol Online Surveys software package. Questions were mainly in the single-best answer format, but some contained free text boxes for more detailed responses. The qualitative data were obtained from the optional free text comment boxes embedded in the questionnaire.

The researchers and a random sample of division of medical education staff at Brighton and Sussex medical school, not directly involved in the research project, performed pretesting of the questionnaires.

### Data management and analysis

Bristol Online Surveys stored completed responses to the questionnaires electronically. The results were transferred to Microsoft Excel and StatsDirect to perform descriptive statistics and the Chi squared test for association, respectively. The qualitative data were analysed manually using a thematic content analysis approach, and was completed independently by two members of the research team (MK and MO), who then discussed findings and agreed on the final themes.

Regarding analysis of the ranking of usefulness data, a score of 1–3 was assigned as being more effective; 4–6 was neutral and 7–9 representing less effective methods.

## Results

### Prescribing lead questionnaire

There were a total of 11 respondents representing 10 out of 15 NHS Hospital Trusts (67 %) contacted. One Trust submitted two responses, of which, one was excluded on the basis that their role with regard to prescribing training was unclear, and was therefore classified as an unreliable source.

Eight out of the 10 NHS Trusts reported that their F1 doctors received induction a week prior to the official start date of their job. 6 NHS Trusts delivered this in the format of a mandatory shadow week that took place before the official start date with the Trust and was considered part of their induction programme. The other 2 NHS Trusts offered this shadow week in a similar format but attendance at this additional component of the induction was not mandatory. One NHS Trust offered induction within the first 4 weeks of the F1s’ start date with their NHS Trust. Overall, 9 out of the 10 NHS Trusts reported that attendance was mandatory at the F1 induction.

Regarding F2 doctor induction, 7 NHS Trusts offered this within 1 week of trainees’ start date. One NHS Trust offered induction within the first 4 weeks of the F2’s start date, and 1 NHS Trust did not know when exactly it was delivered. All 10 NHS Trusts reported that F2 attendance was compulsory at induction.

**Fig. 1:**
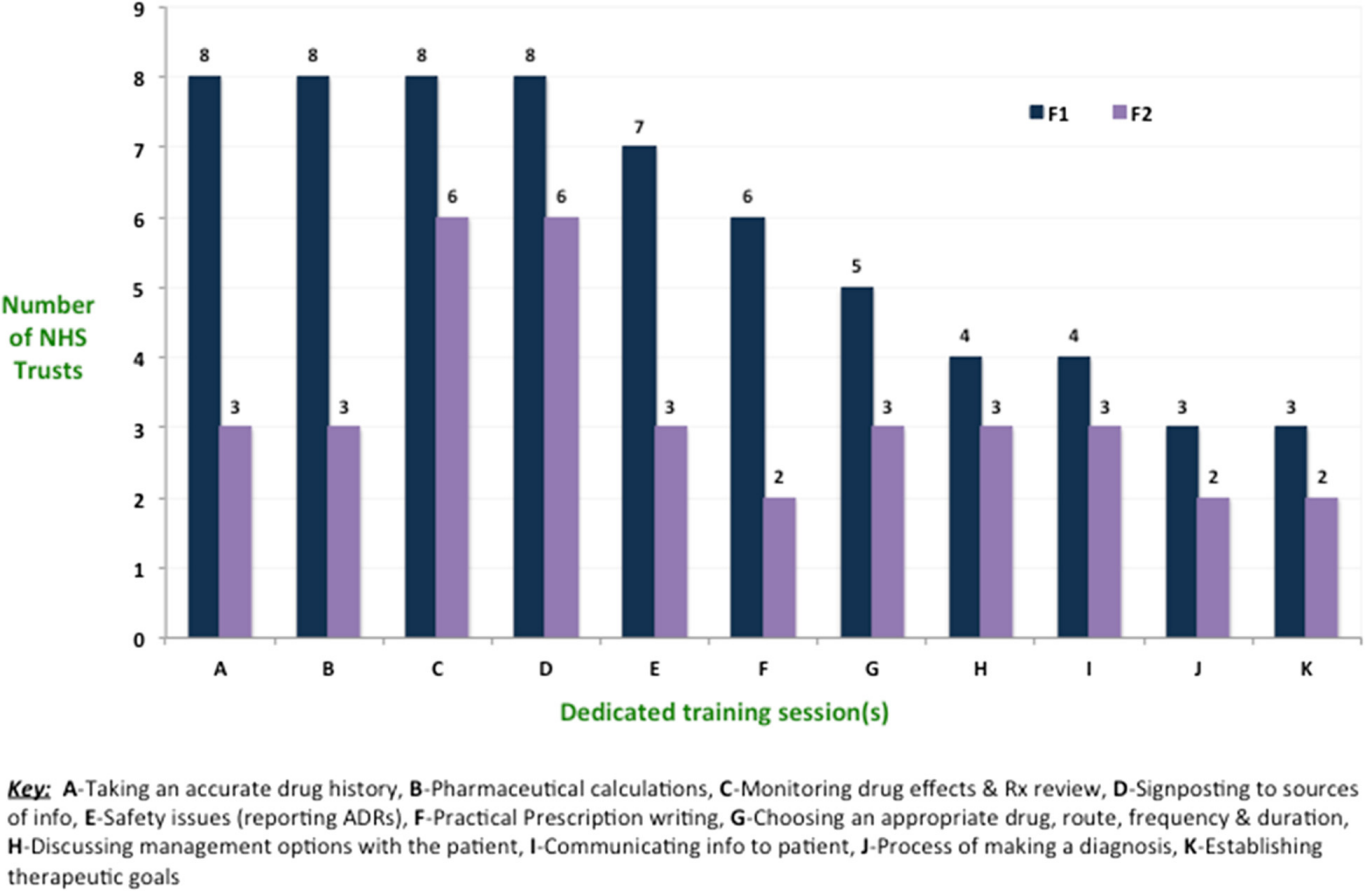
NHS Trusts’ safe prescribing training provisions for F1 and F2 doctors during the 2013–2014 training period in the STFS region

Nine NHS Trusts had a separate induction programme for F1 and F2 trainees and this included specific prescribing training. However, only 5 of the 10 NHS Trusts included a practical prescribing session where trainees got to practice using a drug chart/ e-prescribing system before doing so on the ward. By the end of the foundation training year (including induction), 8 NHS Trusts had provided dedicated session(s) on taking an accurate drug history to F1s, compared with 3 NHS Trusts for F2s. 6 NHS Trusts provided a dedicated practical prescribing session to F1s, while 2 NHS Trusts provided this to their F2s. 8 NHS Trusts provided a dedicated session on pharmaceutical calculations to F1s, while 3 NHS Trusts provided this to F2s. F1s at 7 Trusts received dedicated training on other aspects of safety, but F2s at only 3 Trusts received the same [Fig. 1].

### Foundation doctor questionnaire

**Fig. 2:**
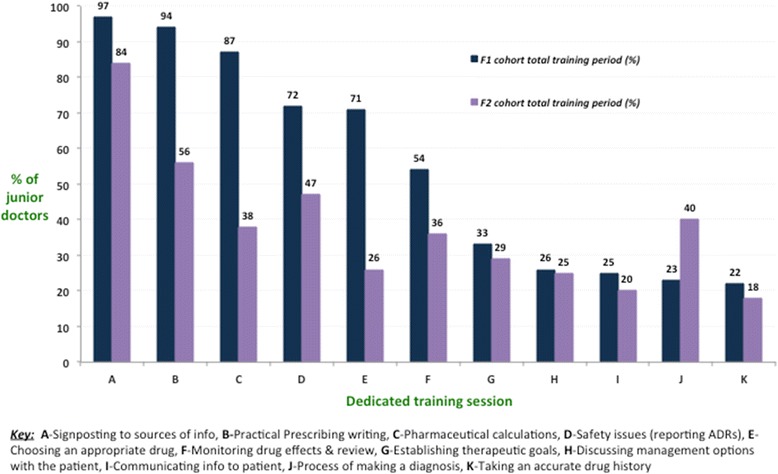
Provision of dedicated safe prescribing training sessions to F1 and F2 doctors during the 2013–2014 training period in the STFS region

A total of 124 foundation trainees (7.2 %) completed the online questionnaire from 16 NHS Hospital Trusts and included 69 F1s (55.6 %) and 55 F2s (44.4 %). 87 % of F1s reported that they received dedicated training in safe prescribing at their NHS Trust induction (*n* = 60, 95 % confidence interval [CI] = 79–95 %) in comparison to 49 % of F2 doctors (*n* = 27, CI = 30–68 %). 80 % of F1s had a practical prescribing session during induction (*n* = 55, CI = 69–91 %), while the corresponding figure for the F2s was 27 % (*n* = 15, CI = 5–49 %). This difference was significant, *X*^*2*^ (1, *N* = 124) = 34.23, *p <0.0001*. By the end of the 1-year training period, which included induction, a total of 94 % of F1s (*n* = 65, 95 % CI = 88–100 %) and 56 % of F2s (*n* = 31, 95 % CI = 39–73 %) had received a dedicated training session in practical prescription writing (including drug chart workshops). This difference was also significant, *X*^*2*^ (1, *N* = 124) = 25.07, *p <0.0001* [Fig. 2].

### Who provides the training?

**Fig. 3:**
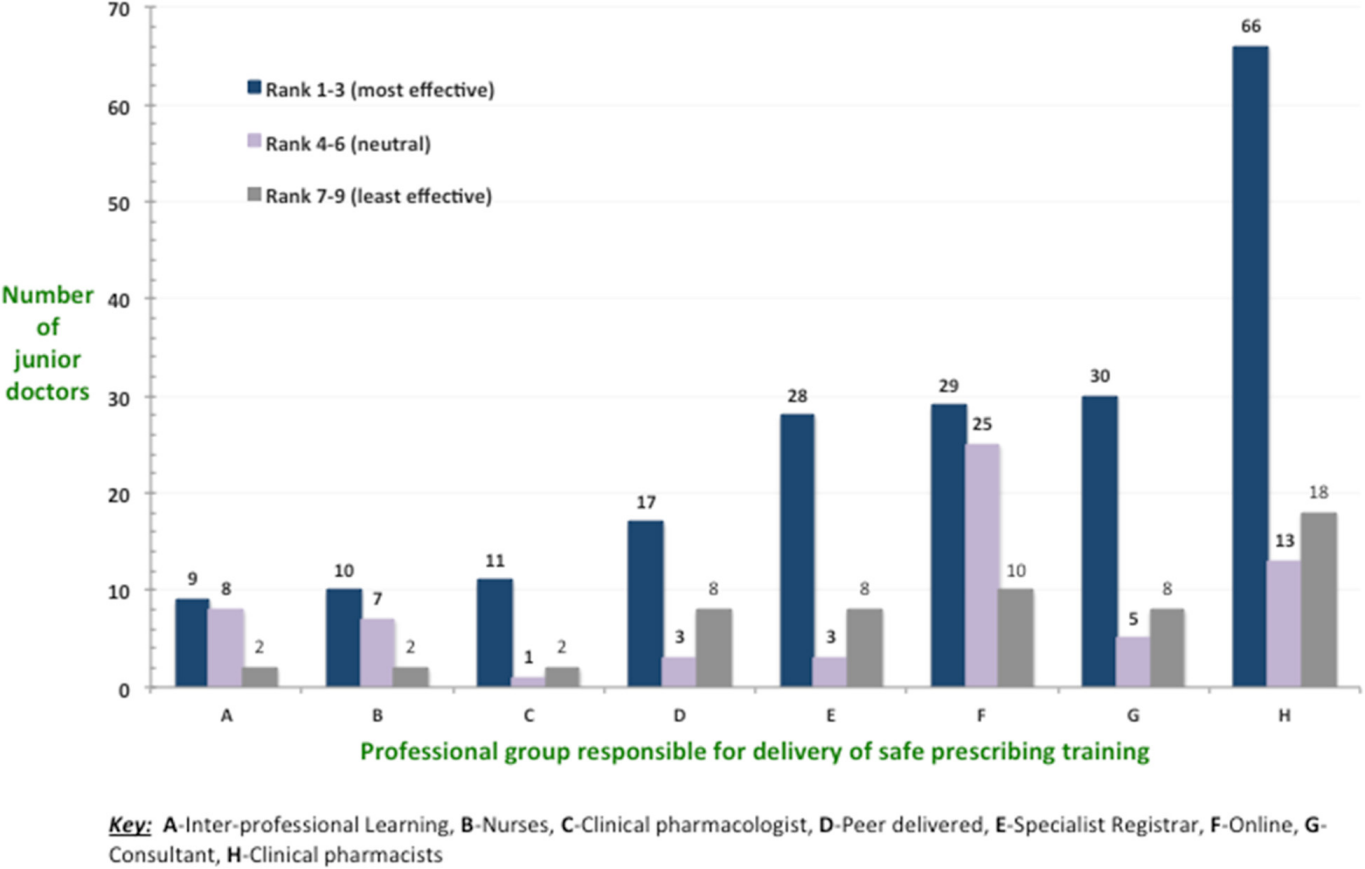
Provision of safe prescribing training by professional group and their relative rank of effectiveness as determined by foundation doctors during the 2013–2014 training period in the STFS region

Seventy eight percent of all foundation trainees (*n* = 97, 95 % CI = 70–86 %) reported dedicated training in safe prescribing by a clinical pharmacist. 55 % completed online training relating to safe prescribing (*n* = 68, 95 % CI = 43–67 %). Clinical pharmacologists provided a dedicated session to just 11 % of foundation trainees (*n* = 14), but received the highest proportion of respondents who found their session(s) very effective 79 % (*n* = 11) [Fig. 3].

### Impact of training on confidence

Ninety percent of F1 doctors reported confidence had improved to some degree by the end of the training period, compared to 65 % of F2 doctors. Only one respondent reported a negative change in prescribing confidence by the end of the training period; this was an F2 doctor.

### Qualitative data

There were 32 doctors’ responses to the optional qualitative question in the trainee questionnaire and a thematic analysis highlighted four emerging themes [Table 1]:

**Table 1:** Thematic analysis results and supporting statements from junior doctors

Theme	Quotes from junior doctor
Importance of medical education as a continuum.	“I feel prescribing training should be provided both in medical school and foundation training. During foundation training it should be regular and not just consigned to induction - some of us don’t work on the ward for our first job.”
	“I think the initial prescribing teaching was good in the induction week however I feel this would be more useful again later on once we were on the wards and prescribing.”
Awareness of importance of safe use of high-risk drugs	“At the end of F1 may be useful to do a session on prescribing in emergencies, small group work so we can relate it to clinical experience.”
	“…I use it to check doses and antibiotic guidelines. The pain section is especially useful.”
	“The new NICE IV fluids guidelines have not been disseminated to us as juniors and I continue to prescribe IV fluids in a relatively arbitrary fashion…. I would also like teaching on analgesia and A&E and on assessing drug charts with polypharmacy”
Working relationship between pharmacists/ pharmacy staff and foundation doctors	“I would love to have practical, relevant teaching on this from a pharmacist - not an anaesthetist, who would view it from a very academic perspective, but from someone with a more practical medicine focus.”
	“In certain posts at Trust X ward rounds are conducted with a pharmacist, which is very useful for specialist prescribing in renal/oncology etc. where we are less familiar. We also have an excellent intranet source of guidance for local deviations from the norm and excellent pharmacists.”
Neglect of F2 doctors in safe prescribing training	“We did not have any formal pharmacy or prescribing induction. This would have been very useful. I think it is presumed that FY2s know this already. This is not always the case.”
	“Ward pharmacists are excellent and should have more dedicated teaching slots for F1s but also F2s as this has been entirely neglected since I became an FY2…”

Importance of medical education as a continuum - 7 out of the 32 responses commented on the importance of the medical education continuum from undergraduate to postgraduate phases, but also on the importance of building on the training received during induction throughout the foundation programme and the need for more training on the whole.

Awareness of importance of training in the safe use of high-risk drugs - 7 of the 32 respondents acknowledged the importance of teaching and learning around high risk drugs such as opioids, antibiotics, anticoagulants, or patient groups who may be more vulnerable to effects of drugs: renal, paediatric, oncology.

Working relationship between pharmacists/pharmacy staff and foundation doctors - this was mentioned by 6 of the 32 participants, and referred to the positive aspects of pharmacists being knowledgeable, accessible and approachable, as well as how patient safety can be affected when these relationships break down.

Neglect of F2 doctors in safe prescribing training was mentioned by 4 of the trainees - all spoke about a lack of dedicated training session’s relating to safe prescribing, and how there are often incorrect assumptions made about F2 knowledge and experience.

## Discussion

This study, for the first time, highlights the difference in safe prescribing training provisions between F1 and F2 doctors. Medical graduates report that they feel ill prepared to safely and competently prescribe medicines [5, 11, 12]. Coupled with findings which indicate higher errors rates in junior doctors [3], it is not surprising to see that NHS Trusts attempt to address this unpreparedness and transition by offering prescribing training to their F1 doctors. However, due to a lack of agreed national or regional standards for the training of junior doctors in safe prescribing, there is little consistency in how various NHS Trusts ensure competencies are achieved [13].

The absence of a standardised programme of the induction provided by NHS Trusts within the region to foundation doctors is concerning, and perhaps more so the fact that one Trust reported that attendance at such a programme was not mandatory. More needs to be done to minimise the stress and anxiety in junior doctors that has been reported during this transition phase [11, 12]. A well-structured induction programme with essential components such as safe practical prescribing training might address this obvious gap.

Results from this study also indicate that a minority of NHS hospital trusts appear to provide dedicated training in making a diagnosis, establishing therapeutic goals, communication, and discussing management options with patients; all processes recognised as being important aspects of the prescribing process [14, 15]. However we noted that the junior doctors themselves indicate that they were well versed in such topics as a result of effective teaching and learning during their undergraduate course. These data corroborate the existing literature that indicates that such tasks were not associated with a feeling of unpreparedness [12]. Arguably, in the light of damaging reports about poor standards of care within the NHS, all tasks associated with the prescribing process should be included in a more formal and visible way in any safe prescribing training programmes irrespective of NHS Trust, i.e. a more standardised content of training. The idea that competency frameworks such as the WHO or BPS guidelines could be used to identify educational interventions for each competency domain has been highlighted [16], and appears to be a logical approach.

Literature documenting the post-graduate teaching and learning of safe prescribing is sparse but there is evidence that learning in the applied setting has been shown to boost prescribing confidence [9]. However, as the association between confidence and competence has not been well established [17], complacency in this area of research is not an option.

It is gratifying to note, from results of this study, that by the end of the training year almost all F1s have had some dedicated training in practical prescribing (94 %) and pharmaceutical calculations (87 %) which are both important aspects of the prescribing process especially at the extremities of age and with impaired physiological function. In comparison, just over a third of F2s receive a dedicated session in these areas by the end of their training year. The timing of the training is also an issue for F2s, in particular, with 36 and 27 % receiving the training at induction for the practical prescription writing and pharmaceutical calculations respectively. The training provided is more purposeful if it occurs earlier during the training year, to support the transition; a period that has been well documented as being an area of concern [11, 12].

Although the transition in medical education from undergraduate to postgraduate is better understood, there is a paucity of data relating to the transition from F1 to F2. There remains uncertainty as to why NHS Trusts do not routinely offer safe prescribing training for F2 doctors, or indeed why the error rate is greatest amongst this cohort. What is clear from this study is that we might be failing to support F2 doctors, who themselves feel that NHS Trusts often make incorrect assumptions about their capabilities: “I think it is presumed that F2s know this already. This is not always the case.” [Table 1]. Potential neglect of the F2 trainees is highlighted in this study.

It has been recommended that sources of drug information are clearly outlined and readily available in clinical workplaces [3]. In order for trainees to have an awareness of such sources of information, appropriate signposting sessions need to be optimised for all trainees. Considering it is not possible to determine how much education is enough, due to variability in how and when people learn, equipping junior doctors with knowledge of the sources of information early in their training is vital. Ideally, such training should be delivered during induction to raise awareness amongst F1 and F2s, before they are required to depend on these sources on the wards, and a more robust and mandatory induction could further support the trainees at the point of transition. The F2s in this study felt that unrealistic assumptions are often made about their knowledge or prescribing experience and therefore they do not receive enough dedicated training in this area of practice. The optimal amount of training in practical prescribing for the F2 cohort of doctors is currently unknown but considering that these doctors have highest prescribing error rate, perhaps more should be done [3].

A number of professionals were involved in the training of foundation doctors in safe and effective practical prescribing. Consultants, specialist registrars, clinical pharmacists and clinical pharmacologists were all perceived to be effective by both F1 and F2s [Fig. 3]. Pharmacists were generally considered as knowledgeable, accessible and important sources of information on safe and effective prescribing by many junior doctors. The relationship with colleagues from pharmacy also emerged as an important theme, with both positive and negative comments being made [Table 1]. In view of the predominant role of pharmacists in this training process and their availability in the clinical setting to further consolidate learning, maintaining the positive aspects of this relationship might be important to nurture prescribing knowledge, attitude and skills.

Despite online learning resources being offered in many Trusts to a significant number of trainees, its perceived effectiveness in this study was unclear. Further investigations as to the reasons for this are required. The benefits of using e-learning in the undergraduate domain have been discussed elsewhere [16, 18], but perhaps its use in the context of postgraduate education is not being optimised.

### Limitations

This study sought to identify the training provisions for safe and effective practical prescribing within the STFS region and not their efficacy. Whilst our data suggest gaps in the provision of safe and effective prescribing training, we have not considered why these gaps are present or how they might affect patient safety. Although there appears to be a positive impact of the training on confidence in prescribing, it would be necessary to establish a link between training provisions and prescribing errors to determine their true value. However there are confounding factors that might make the evaluation of the effectiveness of this process very challenging.

The authors acknowledge that the response rate for the trainees’ questionnaire was low and as a result the sample size of the study is a limitation in terms of its generalisability. A study sampling a greater number of trainees might produce data that highlight the situation nationally. It is worthy of note however, that the majority of NHS Trusts in the region were represented. Furthermore, the fact that the data from both groups (foundation doctors and prescribing leads) gives a consistent insight provides some reassurance that results might reflect the prevailing situation. However, further studies are indicated in order to ensure validity.

## Conclusion

The variation in the teaching and learning of pharmacology and therapeutics in UK medical schools, and an associated lack of emphasis on practical prescribing within UK is well recognised [19]. The current study suggests that a similar trend can be seen in the postgraduate domain.

Despite on-going research in an attempt to reduce prescribing errors it remains a significant problem. One of the fundamentals of therapeutics is the ability to write a clear, legible and safe prescription and most NHS Hospital Trusts do provide some training in safe and effective practical prescribing to their junior doctors, but perhaps a review of the training might optimise the approach and content. NHS Trusts must assume responsibility to ensure that all Foundation doctors feel prepared to undertake this complex task. A more consistent approach across regions in terms of safe prescribing training provisions, with a greater emphasis on the induction period and on F2 doctors might be beneficial.

In spite of the limitations of this study, the results might prompt NHS Hospital Trusts to review their training in safe and effective practical prescribing in early postgraduate medical training. The development of minimum standards for the teaching and learning of safe prescribing during the foundation years of training should be explored. We propose that all NHS Trusts provide training, which includes a practical prescribing session, at induction. In addition, as F1 and F2 needs are slightly different there should be a separate induction process placing emphasis on the more important aspects for each group.

## Additional files

Additional file 1: Trainee questionnaire. (DOCX 309 kb) Additional file 2: Trainer questionnaire. (DOCX 307 kb)

## References

[1] Mucklow J, Bollington L, Maxwell S (2012). Assessing prescribing competence. Br J Clin Pharmacol.

[2] Dean B, Barber N, Schachter M (2000). What is a prescribing error?. Quality in Health Care.

[3] Dornan T, Ashcroft D, Heathfield H, Lewis P, Miles J, Taylor D, Tully M, Wass V. An in depth investigation into causes of prescribing errors by foundation trainees in relation to their medical education. EQUIP study. A report for the GMC 2009 (Online). Available from: http://www.gmc-uk.org/FINAL_Report_prevalence_and_causes_of_prescribing_errors.pdf_28935150.pdf (Accessed July 2015).

[4] Avery T, Barber N, Ghaleb M, Franklin B, Armstrong S, Crowe S, Dhillon S, Freyer A, Howard R, Pezzolesi C, Serumaga B, Swanwick G, Talabi O. Investigating the prevalence and causes of prescribing errors in general practice: The PRACtICe Study (PRevalence And Causes of prescrIbing errors in general practiCe). A report for the GMC 2012 (Online). Available from: http://www.gmc-uk.org/Investigating_the_prevalence_and_causes_of_prescribing_errors_in_general_practice___The_PRACtICe_study_Reoprt_May_2012_48605085.pdf (Accessed April 2014).

[5] Heaton A, Webb DJ, Maxwell SRJ (2008). Undergraduate preparation for prescribing: The views of 2413 UK medical students and recent graduates. Br J Clin Pharmacol.

[6] General Medical Council. Promoting excellence: standards for medical education and training (previously Tomorrow’s Doctors). 2016 (Online). Available at www.gmc-uk.org (Accessed 6 Jan 2016).

[7] The Foundation Programme (2012). The UK Foundation Programme Curriculum 2012 (Updated for 2014).

[8] Tobaiqy M, McLay J, Ross S (2007). Foundation year 1 doctors and clinical pharmacology and therapeutics teaching. A retrospective view in light of experience. Br J Clin Pharmacol.

[9] Rothwell C, Burford B, Morrison J, Morrow G, Allen M, Davies C, Baldauf B, Spencer J, Johnson N, Peile E, Illing J (2012). Junior doctors prescribing: enhancing their learning in practice. Br J Clin Pharmacol.

[10] NHS Health Education England (2016). South Thames Foundation School Prospectus (programmes commencing August 2016).

[11] Tallentire VR, Smith SE, Wylde K, Cameron HS (2011). Are medical graduates ready to face the challenges of Foundation training?. Postgrad Med J.

[12] Illing J, Morrow G, Kergon C, Burford B, Spencer J, Peile E, Davies C, Baldauf B, Allen M, Johnson N, Morrison J, Donaldson M, Whitelaw M, Field M (2008). How prepared are medical graduates to begin practice? A comparison of three diverse UK medical schools.

[13] The National Prescribing Centre (NPC). A single competency framework for all prescribers. May 2012. Available from: https://www.associationforprescribers.org.uk/images/Single_Competency_Framework.pdf (Accessed May 2015)

[14] British Pharmacological Society. Ten Principles of Good Prescribing. Available from: www.bps.ac.uk: (Online); 2010 (Accessed October 2015).

[15] de Vries TPMG, Henning RH, Hogerzeil HV, Fresle DA. Guide to good prescribing, a practical manual. World Health Organisation. Available from: http://apps.who.int/iris/bitstream/10665/59001/1/WHO_DAP_94.11.pdf?ua=1 (Accessed June 2015).

[16] Kamarudin G, Penm J, Chaar B, Moles R (2013). Educational interventions to improve prescribing competency: a systemtic review. BMJ Open.

[17] Ryan C, Ross S, Davey P, Duncan EM, Francis JJ, Fielding S, Johnston M, Ker J, Lee AJ, MacLeod MJ, Maxwell S, McKay GA, McKay JS, Webb DJ, Bond C (2014). Prevalence and causes of prescribing errors: the prescribing outcomes for trainee doctors engaged in clinical training (PROTECT) study. PLoS One.

[18] Maxwell S, Mucklow J (2012). e-Learning initiatives to support prescribing. Br J Clin Pharmacol.

[19] O’Shaughnessy L, Haq I, Maxwell S, Llewelyn M (2010). Teaching of clinical pharmacology and therapeutics in UK medical schools: current status in 2009. Br J Clin Pharmacol.

