# Supplementary material for: Safe prescribing training provision for junior doctors: is this optimal?
Source: BMC Med Educ. 2016 Aug 24;16(1):220. doi: 10.1186/s12909-016-0748-4 (PMC4995635; doi:10.1186/s12909-016-0748-4)
Supplement: Additional file 1: — Trainee questionnaire. (DOCX 309 kb) [file 12909_2016_748_MOESM1_ESM.docx]

Name of NHS Trust: ______________________________________________________________________

At what stage of training in the Foundation programme are you currently? FY1 _____ FY2 _____

When is your Trust induction programme delivered?

Week 1 Week 2 Week 3 Week 4 Month____

Is it compulsory for trainees to attend? Yes /No

Does your induction include specific prescribing training session(s)? Yes / No

Does this include a practical prescribing session with a pharmacist (i.e. do you get to practice using the drug charts/e-prescribing system before doing so on the ward)? Yes/No

What support to aid safe practical prescribing is provided by your Trust during the induction, i.e. shadowing, group interactive sessions, off line support (booklets) etc. _______________________________________________________________________________________________________________________________________________________________________________________________________________________________________________________________________________________

Please indicate which of the following topics have dedicated sessions either in your induction/throughout foundation years. For all that apply please rank how effective these methods were to you becoming a safer prescriber (1 being most useful)?

| ***Task*** | ***Provided*** | ***Usefulness rank*** |
| --- | --- | --- |
|  |  |  |
| Taking an accurate drug history |  |  |
| Process of making a diagnosis |  |  |
| Establishing therapeutic goals |  |  |
| Discussing management options with the patient |  |  |
| Choosing appropriate drug, route, frequency & duration |  |  |
| Practical prescription writing (including drug chart workshops) |  |  |
| Pharmaceutical calculations |  |  |
| Signposting to sources of info (local/national guidelines) |  |  |
| Monitoring drug effects and prescription review |  |  |
| Communicating information to the patient/ carers |  |  |
| Safety issues i.e. how to report suspected or actual ADRs |  |  |
| Other (please specify): |  |  |

How many of the above sessions are mandatory? ___________________________________________________

Who provides this training (tick all that apply) and indicate by order of ranking which you found most effective (1 being most useful)?

| ***Task*** | ***Provided*** | ***Usefulness*** |
| --- | --- | --- |
|  |  |  |
| Consultant |  |  |
| Specialist Trainee |  |  |
| Clinical pharmacist |  |  |
| Clinical pharmacologist |  |  |
| Nurses |  |  |
| Peer delivered |  |  |
| Inter-professional teaching |  |  |
| Online modules/ training |  |  |
| Other (please specify): |  |  |

Is there any extra support relating to safe prescribing during the remainder of the foundation training period? Please give details (i.e. regular training sessions, online modules, personal formulary):

__________________________________________________________________________________________________________________________________________________________________________________________

_____________________________________________________________________________________________

Overall do you feel that the training you received had a big impact on your ability to safely prescribe on the ward on a daily basis? Yes /No

Did your confidence in prescribing safely increased after the training period?

Yes greatly_____ Yes to some extent ______ Yes a little ______ No not at all _____

Depends on the patient/ medicine situation _____

If your Trust ran a prescribing assessment, when was this delivered in relation to induction?

Same day _____ Same week _____ Same month _____

2-3 months later _____ 3-6 months later _____ 6-9 months later _____

>9 months later _____

How soon after the assessment was the feedback provided to you? ____________________________________

What feedback mechanism(s) was employed? _______________________________________________________________________________________________________________________________________________________________________________________________________________________________________________________________________________________

Were you given access to any safe prescribing resources online? Yes/No If Yes, please give details.

_______________________________________________________________________________________________________________________________________________________________________________________________________________________________________________________________________________________

Any Additional comments:

_______________________________________________________________________________________________________________________________________________________________________________________________________________________________________________________________________________________

Notes:

- Questionnaire will be done online to keep carbon footprint to minimum and for ease of completion for participants. Paper copies available upon request.
