# Supplementary material for: Safe prescribing training provision for junior doctors: is this optimal?
Source: BMC Med Educ. 2016 Aug 24;16(1):220. doi: 10.1186/s12909-016-0748-4 (PMC4995635; doi:10.1186/s12909-016-0748-4)
Supplement: Additional file 2: — Trainer questionnaire. (DOCX 307 kb) [file 12909_2016_748_MOESM2_ESM.docx]

Name of NHS Trust: ______________________________________________________________________

Name of person completing: _____________________________________________________________________

Role within organisation: _______________________________________________________________________

When is your Trust induction programme delivered? Week 1 2 3 4 Month FY1 _______ FY2 ______

Is it compulsory for trainees to attend? Y/N

Does your Trust have a separate induction programme for new FY1 and FY2 doctors joining the Trust: Yes/No

What is the duration of your Trust induction programme for FY1’s in total? ___­­_____Hours ________Minutes

What is the duration of your Trust induction programme for FY2’s in total? ___­­_____Hours ________Minutes

At induction, how much time is spent on face-to face teaching sessions?

FY1: ___­­_____Hours ________Minutes

FY2: ___­­_____Hours ________Minutes

At induction, how many hours of online self-directed study are expected of each group?

FY1 ________ FY2: _______

Does your induction include specific prescribing training session(s)? FY1: Yes/No FY2: Yes/No

Does this include a practical prescribing session with a pharmacist (i.e. do trainees get to practice using the drug charts/e-prescribing before they are expected to do so on the ward)? Yes/No

If no, how do you provide support to aid safe prescribing amongst your Foundation doctors during the induction, i.e. shadowing, group interactive sessions, off line support (booklets). For each type listed please indicate how much time is dedicated and if these are mandatory: _______________________________________________________________________________________________________________________________________________________________________________________________________________________________________________________________________________________

__________________________________________________________________________________________________________________________________________________________________________________________

Please indicate which of the following topics have dedicated sessions in your induction/ throughout foundation years. For all those that apply please indicate how many hours are dedicated to each?

| ***Task*** | ***FY1*** | | ***FY2*** | |
| --- | --- | --- | --- | --- |
|  | Induction | Beyond | Induction | Beyond |
| Taking an accurate drug history |  |  |  |  |
| Process of making a diagnosis |  |  |  |  |
| Establishing therapeutic goals |  |  |  |  |
| Discussing management options with the patient |  |  |  |  |
| Choosing appropriate drug, route, frequency & duration |  |  |  |  |
| Practical prescription writing (including drug chart workshops) |  |  |  |  |
| Pharmaceutical calculations |  |  |  |  |
| Signposting to sources of info (local/national guidelines) |  |  |  |  |
| Monitoring drug effects and prescription review |  |  |  |  |
| Communicating information to the patient/ carers |  |  |  |  |
| Safety issues i.e how to report suspected or actual ADRs |  |  |  |  |
| Other (please specify): |  |  |  |  |

How many of the above sessions are mandatory? ___________________________________________________

Who provides this training (tick all that apply) and indicate in hours how much is provided by each person?

| ***Task*** | ***FY1*** | | ***FY2*** | |
| --- | --- | --- | --- | --- |
|  | Induction | Beyond | Induction | Beyond |
| Consultant |  |  |  |  |
| Specialist Trainee |  |  |  |  |
| Clinical Pharmacist |  |  |  |  |
| Clinical Pharmacologist |  |  |  |  |
| Nurses |  |  |  |  |
| Peer delivered |  |  |  |  |
| Inter-professional teaching |  |  |  |  |
| Online modules/ training |  |  |  |  |
| Other (please specify): |  |  |  |  |

Is there any extra support provided relating to safe prescribing during the remainder of the foundation training period? Please give details (i.e. regular training sessions, online modules, personal formulary):

__________________________________________________________________________________________________________________________________________________________________________________________

_____________________________________________________________________________________________

If your Trust runs a prescribing assessment, when is this delivered in relation to induction?

Same day _____ Same week _____ Same month _____

2-3 months later _____ 3-6 months later _____ 6-9 months later _____

>9 months later _____

Is this assessment delivered to both FY1 and FY2 trainees? Yes/ FY1 only/ FY2 only

Are other doctors in the Trust expected to sit the assessment? Yes/No

Who is responsible for conducting the assessment (role)? _____________________________________________

How soon after the assessment is the feedback provided to trainees? ____________________________________

What feedback mechanism(s) are employed? _______________________________________________________________________________________________________________________________________________________________________________________________________________________________________________________________________________________

Are trainees that did not pass the assessment restricted from prescribing on the wards? Yes/ No

What action is taken to support those identified as trainees that require additional assistance in safe prescribing? _______________________________________________________________________________________________________________________________________________________________________________________________________________________________________________________________________________________

Are trainees given access to any safe prescribing resources online? Please give details if Yes. Yes / No

_______________________________________________________________________________________________________________________________________________________________________________________________________________________________________________________________________________________

Any Additional comments:

_______________________________________________________________________________________________________________________________________________________________________________________________________________________________________________________________________________________

Notes:

- Details such as name and position included to facilitate follow up if clarification is required – will not be used for any other purpose.
- Questionnaire will be done online to keep carbon footprint to minimum and for ease of completion for participants. Paper copies available upon request.
